# Native Joint Infections by *Aspergillus* Species

**DOI:** 10.3390/diagnostics11122335

**Published:** 2021-12-11

**Authors:** Christos Koutserimpas, Ifigeneia Chamakioti, Symeon Naoum, Konstantinos Raptis, Kalliopi Alpantaki, George Samonis

**Affiliations:** 1 Department of Orthopaedics and Traumatology, “251” Hellenic Air Force General Hospital of Athens, 11525 Athens, Greece; chrisku91@hotmail.com (C.K.); chamakiotiifigeneia@gmail.com (I.C.); naoumsimeon@gmail.com (S.N.); kraptis1981@hotmail.gr (K.R.); 2 Department of Orthopaedics and Traumatology, “Venizeleion” General Hospital of Heraklion, 71409 Heraklion, Greece; apopaki@yahoo.gr; 3 Department of Internal Medicine, University Hospital of Heraklion, Voutes, 71500 Heraklion, Greece

**Keywords:** fungal septic arthritis, osteoarticular infection, joint infection, *Aspergillus*

## Abstract

Background: Septic arthritis due to *Aspergillus* spp. represents a rare, but severe disease. Nevertheless, clear guidelines regarding most effective medical treatment have not yet been established. The present study is a literature review of all reported cases of fungal septic arthritis due to *Aspergillus* spp, in order to clarify epidemiology, as well as the medical and operative management and their outcome. Methods: A meticulous review of all published septic arthritis infections due to *Aspergillus* has been conducted. Information regarding demographics, causative fungus, antifungal treatment (AFT), surgical intervention, as well as the infection’s outcome were recorded and evaluated. Results: A total of 30 *Aspergillus* spp. strains from 29 hosts have been studied. The patients’ mean age was 45.8 years. The most commonly affected joint was the knee (45.7%), while the predominant symptom was joint pain (62%). Most patients were immunocompromised (72.4%). Diagnosis was established through cultures and/or histopathology. *Aspergillus fumigatus* was the most common responsible fungi (63.3%), followed by *A. flavus* (16.6%) and *A. terreus* (10%). Regarding AFT, the preferred agent proved to be Amphotericin B (14 cases; 48.3%), followed by voriconazole (11; 37.9%), while the mean AFT duration was 5.6 months. In most cases surgical treatment was also performed (in 4 cases no surgery was reported). Treatment was effective in 20 cases (69%). Conclusions: Septic arthritis caused by *Aspergillus* spp. represents a severe clinical entity. It seems that, with the available data, prolonged AFT along with surgical intervention is the preferred management of this infection, while identification of the responsible fungus is of utmost importance.

## 1. Introduction

During the past few years, given that the population of immunosuppressed patients has increased, there has been a dramatic increase in fungal invasive infections, including those caused by *Aspergillus* spp. [1,2,3]. The increased numbers of at-risk immunocompromised patients along with the improved diagnostic techniques through molecular methods have led to an impressive rise of the number of reported fungal infections worldwide [1,2,3].

Invasive Aspergillosis represents important cause of morbidity, as well as mortality in immunosuppressed patients. Fungi of the *Aspergillus* taxon may cause severe infections, such as aspergilloma, exacerbation of asthma, allergic broncho-pulmonary aspergillosis, fungus ball of the sinus, otomycosis, keratitis and endophthalmitis, skin, wound and osteoarticular infections, chronic pulmonary aspergillosis, chronic invasive and granulomatous sinusitis, *Aspergillus* tracheobronchitis, invasive pulmonary aspergillosis, and disseminated disease [3,4]. Approximately 10 million patients per annum are considered at risk to develop invasive aspergillosis and the most frequent species involved is *Aspergillus fumigatus*, followed by *A. flavus* [1,3,4].

Scarce data exist on epidemiology of invasive Aspergillosis and, although *A. fumigatus* continues to be the most common causal mold of this infection, rates of infection due to other emerging species are uncertain [3,4]. Moreover, lack of accuracy in the diagnosis or treatment has led to increased mortality that is ranging between 20% and 100% [4].

Aspergillosis of the respiratory system, as well as soft tissue and skin has been well described. However, osteoarticular infections caused by *Aspergillus* spp., since are rare, are not well understood [1,5].

*Aspergillus* arthritis represents a severe form of invasive aspergillosis. Although, *A. fumigatus* is the most common etiologic agent, being responsible for approximately 80% of the cases, *A. flavus* and *A. terreus* may also cause such infections [6]. *Aspergillus* arthritis arises mainly as hematogenous process in immunosuppressive patients, while as direct inoculation in immunocompetent individuals, such as those undergoing orthopaedic surgery or sustaining trauma [1,6].

Diagnosis can be difficult, due to the fact that presentation of septic arthritis varies widely. Definite diagnosis, achieved by cultures and/or histopathology, after direct sampling, as well as proper therapy are of utmost importance. Causative antifungal treatment (AFT) is required for all such cases, while most of them are in need of surgical intervention [1,6].

There is a scarce of *Aspergillus* septic arthritis cases reported so far in the literature. Hence, guidelines regarding diagnostic methods and techniques, as well as successful treatment are based on limited information.

This is a review of all reported cases of *Aspergillus* septic arthritis presenting epidemiology, patients’ characteristics, as well as medical and surgical management and outcome. The review covers a long-time period. Therefore, during this period diagnostic techniques, as well as medical therapeutic options have changed dramatically.

## 2. Materials and Methods

A thorough electronic search of the PubMed and MEDLINE databases was conducted to identify all published reports associated with cases of *Aspergillus* septic arthritis in native joints through September 2021. Alone and/or in combination, the terms “*Aspergillus* arthritis”, “fungal arthritis”, “*Aspergillus* septic arthritis”, “*Aspergillus* fumigatus arthritis”, “*Aspergillus* joint infection”, “fungal prosthetic joint infection” were searched. In addition, terms including each *Aspergillus* species (e.g., “*Aspergillus terreus* joint infection”, “*Aspergillus flavus* joint infection”, etc.) were also searched. Following the identification of these reports, individual references listed in each publication were further reviewed for ascertainment of additional cases.

The review included articles published in English and only in peer-reviewed periodicals. Articles that fulfilled these criteria but were still excluded were expert opinions, book chapters, studies on animals, on cadavers or in-vitro investigations, as well as abstracts in scientific meetings. Additionally, prosthetic joint infection cases were also excluded. Two cases lacking adequate information were also excluded.

Information regarding age, gender, affected joint, causative *Aspergillus* species, other site of Aspergillosis, C-reactive protein (CRP) and erythrocyte sedimentation rate (ESR) upon presentation, the presence of immunosuppressive medication and/or conditions, type and duration of AFT and surgical intervention. Additionally, the outcome of medical and surgical management, along with the follow-up of each case, were studied. 

It should be noted that the antifungal agent voriconazole was approved in 2003. Since then, it has been regarded as the antifungal agent of choice against infections caused by *Aspergillus* spp. Almost 5 decades have been covered by the present literature review, while voriconazole has been used during the last 2. Therefore, a separate analysis of AFT after 2003 was conducted. Medical treatment has been considered effective, if during the reported follow-up period each patient had no signs and symptoms of the and no recurrence was observed.

All parameters were recorded and analyzed using Microsoft Excel 2019 (Microsoft Corporation, Redmond, WA, USA).

## 3. Results

A total of 29 patients (23; 79.3% males), suffering from septic arthritis due to *Aspergillus* spp., covering a 45-year period (1976–2021), were identified [6,7,8,9,10,11,12,13,14,15,16,17,18,19,20,21,22,23,24,25,26,27,28,29,30,31,32]. The studied population’s mean age was 45.8 years [standard deviation (SD) = 20.5]. (Table 1)

In total, 35 cases of *Aspergillus* spp. septic arthritis were recorded in 29 patients. The affected joint was the knee in 16 cases (45.7%), followed by the shoulder in 5 (14.3%), the ankle in 4 (11.4%), the hip and wrist in 3 each (8.6%), the metacarpal in 2 (5.7%) and the elbow and the sacroiliac joint in 1 each (2.9%). In two patients more than one joint was affected. (Cases 14 and 28 in Table 1). 

Detailed information regarding immunosuppressive conditions, other site of *Aspergillus* infection, as well as symptomatology is presented in Table 1. The predominant symptom was pain, observed in 18 patients (62%), followed by local signs of joint infection, including swelling and high local temperature, in 14 (48.3%), while fever was present in 12 (41.4%).

Furthermore, 21 patients (72.4%) were immunocompromised according to the available information from each report, while 6 of them (28.6%) were organ transplant recipients. Mean C-reactive protein (CRP) and erythrocyte sedimentation rate (ESR) upon initial presentation were 43.9 mg/L (SD = 74.4) and 75.9 mm/h (SD = 35.5) respectively. Another site of *Aspergillus* infection was reported in 8 patients (27.6%). The mean follow-up was found to be 11.8 months (SD = 9.6). 

Regarding imaging methods, magnetic resonance imaging (MRI) was performed in 16 cases (55.2%), followed by plain x-ray in 7 (24.1%), computer tomography in 2 (6.9%) and bone scan in 1 (3.4%). In 7 cases (case 5, 8, 9, 11, 14, 18 and 20 in Table 1) no imaging was reported. 

All cases of *Aspergillus* were diagnosed through cultures and/or histopathology. Galactomannan antigen test was additionally used in case 26 of Table 1, while polymerase chain reaction (PCR) in case 28.

A total of 30 *Aspergillus* spp. strains were isolated from 29 hosts (case 1 in Table 1 yielded both *Aspergillus fumigatus* and *flavus*). Nineteen (63.3%) *Aspergillus fumigatus* were found, followed by 5 (16.6%) *A. flavus* and 3 (10%) *A. terreus*, while 3 (10%) isolates were not further characterized.

Table 2 highlights the management of the reported cases, as well as outcome of the infection. Regarding AFT, 14 cases (48.3%) were treated with a single antifungal regimen, 9 (31%) with two, either simultaneously or consecutively, while 3 (10.3%) were treated with more than 2 antifungal regimens. Information regarding the specific antifungal drug was not reported in 3 cases (10.3%) (cases 8, 18, 24 in Table 2). The mean duration of AFT was found to be 5.6 months (SD = 4.1).

Amphotericin B was the preferred agent in 14 cases [(48.3%), in 5 (35.7%) as monotherapy], followed by voriconazole in 11 cases [(37.9%), in 6 (54.5%) as mono-therapy], itraconazole in 10 [(34.5%), in 3 (30%) as monotherapy], posaconazole and caspofungin in 2 each [(6.9%), none as monotherapy] and micafungin, as well as flucytosine in 1 each [(3.4%), not as monotherapy].

In the present review a total of 17 cases (cases 13–29 in Table 2) have been published after 2003. Therefore, voriconazole was already approved and available as treatment option. During this period, 9 cases (52.9%) were treated with a single antifungal agent, 5 (29.4%) with two, either simultaneously or consecutively, while 2 (11.8%) were treated with more than 2 antifungal regimens. Mean duration of AFT was 5.3 months (SD = 4.2). Voriconazole was the drug of choice in 11 cases [(64.7%), in 6 (54.5%) as monotherapy], followed by amphotericin B in 5 [(29.4%), in 1 (5.9%) as monotherapy], itraconazole in 3 [(17.6%), in 1 (33.3%) as monotherapy], caspofungin and posaconazole in 2 each [(11.8%), none as monotherapy] and micafungin in 1 [(5.9%, not as monotherapy].

During the 1976–2003 period outcome was successful in 6 cases (50%), while mortality rate was 41.7%. Outcome after the initiation of voriconazole was successful in 14 cases (82.4%), while mortality rate was 11.8%. During the whole period of the present study, infection’s outcome was successful in 20 cases (69%), while mortality rate was 24.1%.

Regarding surgical management, in most cases (22; 75.9%) debridement was performed (open or arthroscopically). In 3 cases (10.3%), only arthrocentesis was performed, while in 4 (case 1, 11, 17 and 20 in Table 2) no surgical management was reported.

## 4. Discussion

Fungal septic arthritis represents a rare and severe, even life-threatening, infection. It requires long-term medical, as well as, in most cases surgical treatment [5,30]. Aspergillosis is not a common infection. Its incidence is 12 cases per year/1,000,000 people [33]. Aspergillosis of the respiratory tract, central nervous system, as well as skin and soft tissue structures have been well documented [33]. However, the osteoarticular Aspergillosis is not well described and understood. Data regarding septic arthritis caused by *Aspergillus* spp. are scarce [1,33]. The present study reviewed all septic arthritis cases caused by *Aspergillus* spp., reported so far in the literature, scoping to elucidate epidemiology, patient’s characteristics, causative *Aspergillus* spp., as well as the medical and surgical treatment and their effectiveness. 

The present study reviewed 29 patients affected by 30 strains of *Aspergillus* species, with about 12 months follow-up. These cases cover almost a five-decade period.

*Aspergillus* species are commonly present in soil and decaying matter [5,34]. Invasive Aspergillosis is, most often, reported in immunocompromised hosts [5,34]. In the present review most patients were immunocompromised (72.4%), while almost 30% of them were organ transplant recipients. In cases of organ transplantation, two major points should be kept in mind; if possible, immunosuppressive treatment should be reduced, at least temporarily, since the degree of immunosuppression strongly influences the outcome of invasive aspergillosis. Furthermore, drug interactions must be kept in mind, taking into account the effect of voriconazole that is an agent interfering with the P450 cytochrome oxidase [27]. In particular, voriconazole targets the synthesis of ergosterol biosynthesis by inhibition of the cytochrome P450-dependent enzyme lanosterol 14-alpha-demethylase, resulting finally to severe cell membrane damages and consequently inhibition of the fungal cell growth and replication or death. This drug inhibits also enzymes associated with the function of the P450 cytochrome, regulating the functional respiration chain [27,35].

The studied population was rather young (mean age = 45.8 years), while the male gender was highly represented (males = 79.3%). Another site of *Aspergillus* infection was reported in 8 patients (27.6%). Hence, some immunocompromised patients did not have an apparent pulmonary or other extra-osseous focus, indicating that isolated *Aspergillus* septic arthritis may occur de novo in immunosuppressed hosts, especially in those without prior prolonged antifungal therapy.

The onset of fungal infections is often insidious with non-specific symptoms and, thus, diagnosis is a challenge [24,28,32]. In the reported study sample, the predominant symptom of fungal septic arthritis caused by *Aspergillus* spp. was pain (62%), followed by local inflammation signs (48.3%) and fever (41.4%). Pain, tenderness, edema, and decreased range of motion are common clinical manifestations for most forms of not only fungal but also bacterial arthritis [1]. Furthermore, no other distinct clinical manifestations exist that could reliably distinguish between bacterial and *Aspergillus* septic arthritis. Hence, the constellation of clinical and imaging manifestations of possible joint infection makes necessary the laboratory diagnosis to elucidate the definitive identification of the causative microorganism. Early recognition of *Aspergillus* septic arthritis that plays major role in morbidity and mortality, relies upon the identification of vulnerable populations with symptoms of joint tenderness, pain, local inflammatory signs accompanied with pyrexia. The symptoms of pain and tenderness over a joint in immunosuppressed patients require urgent further evaluation for a septic cause. However, it is of note that also non-immunocompromised patients may be at risk, since previous surgical procedures, can serve as a source of direct inoculation.

The most pathogenic species among *Aspergilli* is *A. fumigatus*, while twenty other species may cause infection [34]. In the present review, *A. fumigatus* was the most common isolated one (63.3%), while *A. flavus* (16.6%) and *A. terreus* (10% each) follow. In all cases of the present study the causative *Aspergillus* strain was isolated from clinical specimens and was identified with cultures and/or histopathology, while galactomannan antigen test and PCR were additionally used in one case each.

Macroscopic and microscopic morphological criteria are the basis of routine identification of the mold in clinical laboratories [3]. Extrolite characterization and multi-locus sequence analysis represents the basis for *Aspergillus* taxonomy [4]. However, in clinical laboratories, these are not performed, while, due to the expansion of DNA sequencing practice, multi-locus sequencing is increasingly used. From the beginning of the last decade, in the clinical literature there are reports of *Aspergillus* isolates identification based on DNA sequencing. This has brought to light the frequent mistaken identification of “cryptic” or sibling species within the sections of *Aspergillus* [4,34,35].

Conventional identification methods of macroscopic, as well as microscopic morphological characteristics by experienced mycologists and identification of molds based on routine DNA sequence are usually not performed in most clinical laboratories. Hence, identification of molds on routine basis may be in most cases imprecise and prone to common errors [4,36,37,38,39]. MALDI-ToF MS now permits routine clinical laboratories to succeed accurate identification of filamentous fungi that previously had been an ability of few laboratories of reference. The critical issue of heterogeneous MALDI-ToF spectra originating from a mixture of cultures of filamentous fungi was addressed successfully by the increase of the number of references that have been obtained from distinct subcultures of strains that were incorporated in the reference spectrum library. This had as result to enhance significantly the ability of identification of filamentous fungi of clinical importance, based on the efficiency of the MALDI-ToF MS [4,39].

The most commonly isolated *Aspergillus* spp. is *A. fumigatus.* The clinical importance of such infections is increasing, since the number of immunocompromised patients is rising [33]. 

Azole resistance represents the fungal strains’ ability to overcome doses of azole compounds that exert effective antifungal activity in other susceptible isolates [3,37,39]. Determination of the minimal inhibitory concentration (MIC) of an antimicrobial agent against a broad range of strains has established the threshold values that distinguish resistant from susceptible strains [37]. MICs distribution along with the pharmacokinetic and pharmacodynamics profiles of a certain antimicrobial allow to determine clinical break points (CBPs), used as predictors to anticipate treatment effectiveness in humans. Different reference methods have been stablished to characterize *Aspergillus* species isolates based on their susceptibility patterns, according to those developed by the European Committee on Antimicrobial Susceptibility Testing (EUCAST) and the Clinical Laboratory Standards Institute (CLSI) [39].

Development of resistance of *A. fumigatus* to antifungal drugs, including the azole compounds, may be an issue in this infection’s management [5,34,35,36,37]. Due to extensive use of azoles as pesticides in the Netherlands and the United Kingdom, an increased rate of azole resistance has been reported in these countries. However, the prevalence of such a resistance has been shown to remain low in other countries [35]. Hence, it is most important to identify the strain, its susceptibilities and to obtain accurate MIC values following the *Aspergillus* isolation. It is important to note from 2020 the EUCAST has standardized Antifungal Clinical Breakpoints for a number of methods measuring MICs. Nevertheless, it should be noted that the immune condition of the host is the most critical issue regarding the success of treatment [38,39].

Triazoles and amphotericin B are the antifungal agents recommended for treatment of invasive Aspergillosis, possessing cidal action, while echinocandins may be useful, possessing only static effect. Prolonged AFT is required for the management of such infections. In 2003, voriconazole was introduced and since then this agent has been proved the drug of choice [40]. Voriconazole has all the characteristics of azole compounds, such as moderate hepatotoxicity and minor nephrotoxicity as compared to all amphotericin formulations [37,40].

Voriconazole is a triazole with a broad spectrum of antifungal activity. Its efficacy against all *Aspergilli* of clinical importance represents this drug’s most important merit. It has been shown to be the most active and the first-line antifungal agent for the medical management of invasive Aspergillosis [40]. Voriconazole is also effective for a number of other fungal infections, such as candidemia in patients with neutropenia, those with esophageal and/or disseminated candidiasis. It is recommended as first-line therapy for severe mycoses, such as those caused by *Scedosporium* and *Fusarium* species. The drug is not effective for the treatment of mucormycoses, caused by the species *Rhizopos*, *Mucor* and *Absidia*. Voriconazole has been shown to possess non-linear pharmacokinetics and its dose-response relationship exhibits a wide variability among different patients [40]. The therapeutic index is narrow and is important to be noted that serum concentrations are significantly influenced by a broad range of other drugs due to extensive drug to drug interactions. Voriconazole is available as oral and intravenous preparations [3,40].

The results of the present study have shown that, amphotericin B has been used in 14 cases (48.3%), either as monotherapy or in combination with some other antifungal drug, while voriconazole in 11 (37.9%), either as monotherapy or in combination with some other antifungal. The present study covers a very long period. However, the results after 2003 have shown that voriconazole was the most used agent [11 cases (64.7%)], while the use of amphotericin B was limited [5 (29.4%)]. The mean duration of AFT was 5.6 months.

Amphotericin B is a very broad-spectrum antifungal drug used for decades. However, it is characterized by considerable toxicity. Amphotericin B is nephrotoxic and may cause severe electrolyte disturbances These side effects may limit its use, especially when needs to be given for a long period [38,41]. The liposomal formulations of amphotericin B are less nephrotoxic, but if given for prolonged period may damage the kidneys’ function [41]. In the present study exact information about the amphotericin B type could not be extracted from the given data in the majority of the cases. However, it is reasonable to assume that Amphotericin B lipid or liposomal formulations have been the drugs of choice during the last 2 decades, since during this period the therapeutic use of deoxycholate amphotericin B has been abandoned.

Fungal septic arthritis also requires in most cases surgical treatment. In the present study, in the majority of patients (75.9%), debridement was performed. The severity of the disease is depicted through the mortality rate. The overall mortality rate was found to be 24.1%. However, after 2003 when voriconazole was introduced, mortality has dropped to 11.8%.

The present study covers a period of almost 5 decades. During this time antifungal treatments, as well as the knowledge about fungal cell biology have changed dramatically. These facts, along with unavailability of some information from a number of the reviewed cases represent limitations of the study. Hence, dosages, drug serum-levels, MICs and side effects of the used antifungal agents, in some cases were not described. Nevertheless, this review provides valuable information about epidemiology, symptomatology, treatment, as well as outcome of cases of septic arthritis caused by *Aspergillus* spp. occurring for 5 decades.

In conclusion, septic arthritis caused by *Aspergillus* spp. is a severe and, in some cases, life-threatening infection, requiring early diagnosis and prompt multidisciplinary treatment. Often, surgical debridement becomes necessary. Prolonged AFT, guided by susceptibility tests, along with surgical intervention represent the optimal current therapeutic approach. Furthermore, in cases of septic arthritis proven culture negative for bacteria and/or cocci, high index of suspicion for fungal pathogens should be present, especially in immunocompromised patients. Since these infections are rare, physicians should be trained to promptly recognize them through studies featuring their symptoms, severity, treatment and outcome.

## Figures and Tables

**Table 1:** Patient’s demographics, responsible fungus, affected joint, immunosuppressive condition and/or medications, other site of Aspergillosis and symptoms. M: male, F: female, RT: recipient of renal transplant, IST: immunosuppressive treatment, AML: acute myelogenous leukemia, DM: diabetes mellitus, LSI: local signs of inflammation.

Case No	Year	Authors	Gender/ Age	*Aspergillus* Species	Affected Joint	Immunosuppressive Conditions and/or Medication	Other Site of Aspergillosis	Symptoms
1.	1976	Prystowsky et al. [7]	M/1.5	*A. flavus, A. fumigatus*	Knee	Idiopathic aplastic anemia	Lung, Skin	-
2.	1993	Motte et al. [8]	M/69	*A. fumigatus*	Knee	-	Vascular graft	Pain, LSI
3.	1995	Alvarez et al. [9]	M/29	*A. fumigatus*	Knee	RT, IST	-	Pain, LSI, Pyrexia
4.	1997	Steinfeld et al. [10]	M/51	*A. terreus*	Knee	Liver cirrhosis	-	LSI, Pyrexia
5.	1997	Steinfeld et al. [10]	M/69	*A. fumigatus*	Knee	-	Vascular graft	Pain, LSI
6.	1997	Garcia-Porrua et al. [11]	F/58	*A. fumigatus*	Knee	AML, chemotherapy		Pyrexia
7.	1999	Gunsilius et al. [12]	M/59	*A. fumigatus*	Wrist	Acute lymphoblastic leukemia, chemotherapy	Aneurysm of the arteria cerebri	Pain, LSI
8.	2001	Panigrahi et al. [13]	M/35	*A. fumigatus*	Knee	IST	-	Pain, LSI
9.	2001	Panigrahi et al. [13]	M/45	*A. fumigatus*	Knee	AML, chemotherapy	Lung	Pain
10.	2002	Kaneko et al. [14]	F/57	*A. fumigatus*	Knee	Recipient of liver transplant, IST	Lung	-
11.	2003	Stratov et al. [15]	M/42	*A. flavus*	Wrist	-	-	-
12.	2003	Bodur et al. [16]	M/17	*A. fumigatus*	Knee	Chronic granulomatous disease	-	Pyrexia
13.	2004	Saba et al. [17]	M/57	*A. fumigatus*	Elbow	AML	-	LSI, pyrexia
14.	2004	Mekan et al. [18]	M/18	*A. fumigatus*	Knee (bilateral), Ankle (Bilateral), Metacarpal (bilateral)	Chronic granulomatous disease	-	Pain, LSI, fatigue, pyrexia
15.	2004	Sohail et al. [19]	M/88	*A. fumigatus*	Shoulder	-	-	Pain, LSI
16.	2004	Lodge et al. [20]	M/64	*A. fumigatus*	Ankle	Recipient of lung transplant, IST	-	Pain, LSI, pyrexia
17.	2005	Mouas et al. [21]	M/46	*A. terreus*	Wrist	-	-	-
18.	2006	Kumashi et al. [22]	M/18	spp.	Knee	Hematologic malignancy	-	-
19.	2007	Ersoy et al. [23]	Μ/34	*A. fumigatus*	Ankle	RT, IST, chronic hepatitis	-	Pain, LSI
20.	2009	Horn et al. [24]	F/60	*A. fumigatus*	Shoulder	RT, recipient of pancreatic transplant, IST	Lung, Skin	-
21.	2010	Yu et al. [25]	M/18	*A. flavus*	Shoulder	Acute lymphoblastic leukemia, chemotherapy	Lung	Pain, LSI, pyrexia
22.	2011	Golmia et al. [26]	F/58	*A. fumigatus*	Sacroiliac joint	Diffuse systemic sclerosis, autologous stem cell transplant	-	Pain
23.	2012	Figuères et al. [27]	M/43	*A. fumigatus*	Hip	RT, recipient of pancreatic transplant, IST	-	-
24.	2012	Sun et al. [28]	M/23	spp.	Knee	-	-	Pain, LSI, pyrexia
25.	2012	Hall et al. [29]	F/72	*A. fumigatus*	Shoulder	DM, coronary artery disease	-	Pain
26.	2014	Tiwari et al. [30]	M/28	*A. flavus*	Knee	-	-	Pain, LSI, pyrexia
27.	2015	Yoon et al. [6]	F/49	spp.	Hip	-	-	Pain
28.	2016	Kumar et al. [31]	M/60	*A. flavus*	Hip, Knee	DM	-	Pain, pyrexia
29.	2021	Liu et al. [32]	M/61	*A. terreus*	Shoulder	DM, end stage renal disease	-	Pain, pyrexia

**Table 2:** Antifungal treatment (AFT), duration of AFT, Surgical intervention, follow-up and infection’s outcome are presented. (*): death due to infection.

Case No	AFT	Total Duration of AFT (Months)	Surgery	Follow-Up (Months)	Outcome
1.	Amphotericin B	1	-	1	Failure *
2.	Itraconazole	9	Arthroscopic debridement	24	Success
3.	Amphotericin B	2	Debridement	3	Failure *
4.	Itraconazole	4	Arthroscopic debridement	3	Success
5.	Amphotericin B, itraconazole	9	Arthroscopic debridement	36	Success
6.	Amphotericin B, itraconazole	-	Debridement	26	Success
7.	Amphotericin B, itraconazole	10	Debridement		Failure *
8.			Arthrocentesis		Failure *
9.	Amphotericin B		Arthrocentesis		Failure *
10.	Amphotericin B, 5-fluorocytosine, itraconazole	12	Arthrocentesis	12	Success
11.	Amphotericin B	2	-	12	-
12.	Amphotericin Β, itraconazole	6	Debridement	4	Success
13.	Amphotericin B, itraconazole	5	Debridement	5	Failure *
14.	Itraconazole	6	Debridement	6	Success
15.	Voriconazole	9	Debridement	9	Success
16.	Itraconazole, amphotericin B, posaconazole	12	Debridement	24	Success
17.	Voriconazole	3	-		Success
18.			Debridement		-
19.	Amphotericin B	1.5	Debridement	12	Success
20.	Voriconazole, Posaconazole, micafungin	2.3	-	2,5	Failure *
21.	Caspofungin, voriconazole	4	Arthroscopic debridement	1	Success
22.	Caspofungin, voriconazole	2	Debridement		Success
23.	Voriconazole	6.5	Debridement and femoral head resection	7	Success
24.	-		Debridement	12	Success
25.	Voriconazole	16	Debridement	24	Success
26.	Amphotericin B, voriconazole	2	Debridement	3	Success
27.	Amphotericin B, voriconazole	6	Arthroscopic debridement	19	Success
28.	Voriconazole	2	Arthroscopic debridement	15	Success
29.	Voriconazole	2	Debridement	12	Success

## Data Availability

Not applicable.
